# Patient Selection and Maintenance of Standards to Improve the Safety of Immediate Sequential Bilateral Cataract Surgery in Tropical Regions

**DOI:** 10.7759/cureus.91356

**Published:** 2025-08-31

**Authors:** Gede Pardianto, Ruth Anastasia, Diyah Purworini

**Affiliations:** 1 Department of Ophthalmology and Vision Sciences, Universitas Prima Indonesia, Medan, IDN; 2 Department of Biomedical Sciences, Universitas Prima Indonesia, Medan, IDN

**Keywords:** bilateral, immediate, maintained standard, patient selection, phacoemulsification, safety, sequential, tropical region

## Abstract

Purpose: To determine the patient selection and maintain standards to improve the safety of immediate sequential bilateral cataract surgery (ISBCS) in tropical regions that are hot, humid, and prone to infections.

Materials and methods: Thirty-nine participants (78 eyes) meeting stringent inclusion criteria underwent ISBCS. The stricter criteria included absence of systemic disease, absence of ocular abnormalities, and presence of physiological cataract (not traumatic cataract, complicated cataract, pseudoexfoliation, etc.). ISBCS was performed using the phaco-chop technique with a Venturi phaco machine, bimanual irrigation/aspiration, and hydro-implantation of the intraocular lens (IOL). Intraoperative complications (posterior capsule rupture (PCR) and vitreous loss) and postoperative outcomes (corneal edema, toxic anterior segment syndrome (TASS), and endophthalmitis) were monitored. Uncorrected visual acuity (UCVA), best-corrected visual acuity (BCVA), and intraocular pressure (IOP) were measured and compared pre- and postoperatively. Statistical analysis was performed, with p < 0.05 considered statistically significant.

Results: No intraoperative complications (PCR and vitreous loss) were observed. And no postoperative (visual-disturbing corneal edema, TASS, and endophthalmitis) cases were observed. Minimal corneal edema was confirmed with no negative impacts on visual functions. Significant improvements occurred in UCVA and BCVA (p < 0.001), respectively, alongside significant reductions in IOP (p < 0.001).

Conclusion: Strict patient selection and maintained standards may improve the safety of ISBCS in tropical regions by preventing intraoperative PCR and vitreous loss, as well as postoperative complications such as visual-disturbing corneal edema, TASS, and endophthalmitis. This approach may also facilitate rapid rehabilitation of UCVA and BCVA, maintain corneal stability, and reduce IOP.

## Introduction

Cataracts remain a leading cause of treatable blindness globally. Phacoemulsification, a minimally invasive technique, has revolutionized cataract treatment by enabling safety, less pain, and rapid visual recovery [1]. Despite its safety profile, complications such as posterior capsule rupture (PCR), vitreous loss, corneal edema, toxic anterior segment syndrome (TASS), and endophthalmitis necessitate meticulous surgical protocols [1,2].

Immediate sequential bilateral cataract surgery (ISBCS) offers operational and cost-effective advantages, particularly in resource-limited settings. With some refinements in safety protocols, the rate of ISBCS tends to increase. However, its safety in tropical climates, where weather, humidity, elements, and infection risks are way elevated, requires further validation [3-5]. The complications that have long sparked debate over performing ISBCS will, of course, increase sharply if carried out in tropical regions [6,7].

Considering the need on the one hand and concerns on the other, further research with high-standard surgery and strict patient selection must be carried out [5]. The safety of surgery is the ultimate goal to ensure surgery’s good results, including fine visual outcomes. Thus, studies on factors that can improve the safety of ISBCS in tropical regions are highly important and make a significant contribution to efforts to eliminate cataract-related blindness.

## Materials and methods

This study employed a prospective quasi-experimental pre-post intervention design at Sabang-Merauke Eye Center (SMEC), Medan, Indonesia, spanning August 2024 to June 2025. A total of 78 eyes of 39 participants were enrolled and underwent ISBCS following comprehensive and meticulous patient selection.

The stringent inclusion criteria included absence of systemic disease, absence of ocular abnormalities, and presence of physiological cataract (not traumatic cataract, complicated cataract, pseudoexfoliation, etc.), with best-corrected visual acuity (BCVA) worse than 20/70, anatomically normal ocular structures (including conjunctiva, cornea, and retina), and intraocular pressure (IOP) below 21 mmHg. Patients who were anxious both before and during the surgery were excluded.

Surgical maintenance standards included optical biometry performed using the IOLMaster 500 (Carl Zeiss Meditec, Dublin, CA) for IOL power calculations. Before the surgery, the patient’s eyes were instilled with 0.5% tetracaine hydrochloride eye drops (Pantocain, PT Cendo, Indonesia) for topical anesthesia and were well dilated with 1% tropicamide eye drops (Mydriatil, PT Cendo, Indonesia). All equipment and instruments used for the operation were sterilized. All instruments used extraocularly, such as speculums, were immersed in a 5% povidone-iodine solution. Skin and eyelid disinfection in the surgical field was performed using 10% povidone-iodine, while conjunctival disinfection was done with 5% povidone-iodine. All patients underwent meticulous and sterile phacoemulsification performed by a single surgeon (GP) using the Stellaris Elite Venturi phaco machine (Bausch+Lomb, Bridgewater, NJ). The procedure utilized the phaco-chop technique, bimanual irrigation/aspiration, and IOL hydro-implantation. At the conclusion of the surgery, strict intracameral administration of 0.5% levofloxacin (Optiflox, PT Erela, Indonesia) was employed for infection prophylaxis. The procedure took about four to eight minutes for each eye.

The strict standard of postoperative management included detailed education and instructions to protect the eyes from exposure to water, wind, dust, smoke, and other substances that may irritate the eyes and carry microbes. Patients were provided with protective glasses and an eye shield to wear while sleeping. Additionally, patients were given 0.5% levofloxacin eye drops (LFX, PT Cendo, Indonesia) and 0.1% sodium diclofenac eye drops (Noncort, PT Cendo, Indonesia), each to be instilled one drop six times a day. No oral or systemic medications were given. Patients were asked to return for follow-up on the first day and seven days postoperatively for evaluation.

Outcome monitoring focused on intraoperative complications (PCR and vitreous loss) and postoperative complications (visual-disturbing corneal edema, TASS, and endophthalmitis), alongside comparisons of uncorrected visual acuity (UCVA), BCVA, and IOP between preoperative and one-month postoperative assessments.

VA is described in decimals, IOP in mmHg, with a 95% confidence interval. Statistical analysis was conducted using IBM SPSS Statistics for Windows, Version 29.0.2.0 (Released 2022; IBM Corp., Armonk, New York, United States) with paired t-tests applied to determine significance (p < 0.05). This study meets the Declaration of Helsinki [8], and the SMEC Hospital Research Ethical Review Committee approved the study (Approval Number EA00000672, August 21, 2024).

## Results

A total of 78 eyes of 39 participants underwent maintained-standard ISBCS following previous meticulous patient selection. The study population had a mean age of 65.74 years with a standard deviation of 14.58 years, comprising 18 (46.15%) male and 21 (53.85%) female participants. Ethnic distribution analysis revealed 21 (53.85%) Malay participants, 15 (38.46%) Chinese participants, and three (7.69%) Indian participants (Table 1). Regarding surgical safety outcomes, no complications were observed, with zero cases of intraoperative PCR and vitreous loss, as well as postoperative visual-disturbing corneal edema, TASS, and endophthalmitis (Table 2).

**Table 1:** Baseline characteristics Sex and race are presented as number (percentage).

Characteristics	Numbers (%)
Sex	
Male	18 (46.15%)
Female	21 (53.85%)
Race	
Malay Indonesian	21 (53.85%)
Chinese Indonesian	15 (38.46%)
Indian Indonesian	3 (7.69%)

**Table 2:** Comparison of VA and IOP following ISBCS UCVA and BCVA are presented as decimal values with standard deviation (SD) and range (minimum to maximum), while IOP is presented in millimeters of mercury (mmHg) with SD and range (minimum to maximum). UCVA: uncorrected visual acuity; BCVA: best-corrected visual acuity; ISBCS: immediate sequential bilateral cataract surgery; IOP: intraocular pressure

VA and IOP	Preoperative	One-month postoperative	P-value
VA (decimal)			
UCVA	0.118 ± 0.061 (0.016-0.25)	0.853 ± 0.084 (0.50-1.0)	<0.001
BCVA	0.232 ± 0.042 (0.016-0.285)	0.941 ± 0.051 (0.80-1.0)	<0.001
IOP (mmHg)	18.093 ± 1.519 (16-20)	12.871 ± 1.401 (11-15)	<0.001

Visual acuity (VA) and IOP measurements demonstrated significant improvements following the procedure (Table 2). Following minimal corneal edema, UCVA showed marked enhancement, increasing from 0.118 ± 0.061 (0.016-0.25) preoperative to 0.853 ± 0.084 (0.50-1.0) postoperative (p < 0.001). Similarly, BCVA exhibited substantial improvement, rising from 0.232 ± 0.042 (0.016-0.285) to 0.941 ± 0.051 (0.80-1.0) with p < 0.001.

IOP measurements revealed a significant reduction from baseline values of 18.093 ± 1.519 (16-20) mmHg to postoperative levels of 12.871 ± 1.401 (11-15) mmHg (p < 0.001), following the surgical intervention.

## Discussion

ISBCS combines the efficiency of phacoemulsification with bilateral intervention, reducing patient burden and healthcare costs [3-5]. Additionally, phacoemulsification has many complications both intra- and postoperatively [6,7,9]. PCR and vitreous loss are intraoperative complications that should be avoided [10]. Corneal edema, TASS, and endophthalmitis are among postoperative complications that must be watched out for and avoided [11-15].

Unsafe implementation of ISBCS can lead to adverse effects and problems for vision [6]. Therefore, a well-prepared and thorough plan is required [4,5,7,9]. The safety of ISBCS in tropical regions with their climates, where weather, hot environment temperature, humidity, elements, and infection risks are way elevated, needs to be emphasized [1].

This study implemented two crucial measures: firstly, selecting the patients; and secondly, upholding standards in the execution of ISBCS. Patient selection involved the application of strict inclusion criteria prior to performing ISBCS. The stricter criteria included absence of systemic disease, absence of ocular abnormalities, and presence of physiological cataract (not traumatic cataract, complicated cataract, pseudoexfoliation, etc.). If the patient appears anxious, they will be excluded from the study, and for safety reasons, ISBCS will not be performed. Maintained-standard ISBCS was performed using the careful phaco-chop technique with a Venturi phaco machine, bimanual irrigation/aspiration, and IOL hydro-implantation with a strict intracameral antibiotic for infection prophylaxis that was administered at the conclusion of the surgery.

Additional standards in this study that may differ from the existing standards for ISBCS elsewhere include: immersing all instruments used extraocularly during the surgery, such as speculums, in 5% povidone-iodine, conjunctival disinfection using 5% povidone-iodine, intracameral injection of 0.5% levofloxacin at the end of the surgery, strict postoperative explanation and education to patients to avoid water, wind, dust, smoke, and any substances that may irritate and infect the eyes, providing patients with protective glasses and eye shields, as well as administering antibiotic and anti-inflammatory eye drops, all of which are expected to reduce the incidence of infection in tropical areas. Additionally, in this study, a safe surgical technique was chosen, performed by a single experienced surgeon, using a Venturi phaco machine, with bimanual irrigation/aspiration and IOL hydro-implantation to minimize the occurrence of intraoperative complications. The procedure takes about four to eight minutes for each eye. The above-mentioned aspects are some of the strengths of this study that are hoped to be adopted over time in any other tropical locations and by different surgeons.

The absence of complications in this cohort aligns with studies emphasizing strict patient selection, meticulous sterile protocols, high-standard surgery, and strict postoperative management [4,5,7,9]. The absence of intraoperative complications (PCR and vitreous loss) assures the safety of the maintained-standard ISBCS that was performed using the careful phaco-chop technique with a Venturi phaco machine, bimanual irrigation/aspiration, and IOL hydro-implantation. Patient selection also played a role in ensuring the safety of ISBCS in this study. The safety finding in this study is consistent with the findings of several previous studies [4,5,7,9,16].

The absence of postoperative complications (visual-disturbing corneal edema, TASS, and endophthalmitis) in this study indicates that patient selection and maintained standards, supported by sterilization protocols, sterile and high-standard surgery, intracameral antibiotic administration, and postoperative management, can enhance the safety of ISBCS in tropical regions that are hot, humid, and prone to infection. This is consistent with the findings of several previous research studies [17,18].

Minimal corneal edema in this study’s ISBCS guarantees good visual acuity. VA improvement in this study is an indication that the procedure is safe and complication-free [1,4,5,7,9,16]. Postoperative IOP reduction may reflect anterior chamber angle widening from in-the-bag IOL placement, potentially safeguarding long-term retinal nerve fiber layer (RNFL) integrity, because high IOP is a potential risk for RNFL damage, and vice versa [1,19].

Although a number of clinics have already switched to ISBCS during the COVID pandemic, in some regions, ISBCS is not recommended due to complications and infection concerns [10,17]. Regarding that particular concern, strict patient selection is mandatory. High surgical standards are maintained in all aspects, including preoperative examination, operating room and instrument sterilization, disinfection, intracameral antibiotic administration, and postoperative management [1,17,18]. Safe and careful surgery and its techniques are crucial. All ISBCS procedures must adhere to the guidelines from various bilateral cataract surgery societies around the world [4,5,7,9]. ISBCS and phacoemulsification should be performed with greater caution in challenging cases. It is recommended that an experienced surgeon perform ISBCS [18,20-22]. The use of intracameral antibiotics and postoperative antibiotic eye drops is mandatory and non-negotiable in ISBCS to avoid any unwanted infections, let alone in tropical areas [1,17,18]. A limitation of this study is the relatively small sample size. Further studies with a bigger sample size and a larger multicenter study are required to ensure the safety of ISBCS.

## Conclusions

Strict patient selection and maintained standards may improve the safety of ISBCS in tropical regions by preventing intraoperative PCR and vitreous loss, as well as postoperative complications such as visual-disturbing corneal edema, TASS, and endophthalmitis. This approach may also facilitate rapid rehabilitation of UCVA and BCVA, maintain corneal stability, and reduce IOP.
